# English goalkeepers are not responsible for England’s poor performance in penalty shootouts in the past

**DOI:** 10.1038/s41598-021-04118-6

**Published:** 2021-12-28

**Authors:** Michel Brinkschulte, Philip Furley, Maximilian Klemp, Daniel Memmert

**Affiliations:** Institute of Training and Computer Science in Sport, German Sport University Cologne, Am Sportpark Müngersdorf 6, 50933 Köln, Germany

**Keywords:** Psychology, Human behaviour

## Abstract

Scrutinizing public opinion is one of the central goals of science as the divergence between public opinion and scientific evidence can have negative consequences. The present study aims to further investigate the alleged English ‘penalty curse’ and determine if it can be linked to the prevalent stereotype of the ‘English goalkeeper problem’. We analyzed a large sample of 2379 penalty kicks that 629 different goalkeepers faced in World Cups and European Championships, as well as in the Champions and Europa League by comparing the goalkeeper success rates of different nations by fitting a generalized linear model (binomial regression) to the data. However, the results do not reveal meaningful differences between the success rates (on average 22.23%). Consequently, we conclude that English goalkeepers are not responsible for England’s poor performance in penalties in the past as they perform as well as goalkeepers from other nations and, in turn, provide a counterargument to the widespread stereotype that ‘England has a goalkeeper problem’.

Scientific findings do not always reflect the opinion of the public. For example, while there is clear scientific evidence that human activities cause global warming, parts of the public do not necessarily believe in these findings^1^. This divergence between public opinion and scientific evidence can have negative consequences. In the case of global warming, it seems obvious that it is problematic if large proportions of the public do not believe that their behavior and activities contribute to global warming. Beyond environmental issues, there are certainly many more examples of problems in people’s everyday lives that can occur if public opinion deviates from scientific knowledge. If large proportions of people hold certain stereotypes concerning different national or ethnic groups, for instance, this can lead to discrimination or stereotype threat (i.e., people behave/perform in accordance with the stereotype^2^). For this reason, it is important to conduct rigorous scientific tests to find out if public opinion stands the test of the scientific method.

Given the immense public interest in professional sports, it is not surprising that there are many public opinions or that stereotypes exist in sports (e.g.,^3^). For example, a recent study^4^ did not find evidence for the wildly held stereotype that English football players are bad at taking penalties. Nor did this research find evidence for other commonly held stereotypes, for instance, that players of some nations like Germany perform extraordinarily well in penalty kicks. Pertinent to the present research, there is another commonly held stereotype concerning English and German soccer players, and that is that English goalkeepers are ‘no good at goalkeeping’ while German goalkeepers are exceptionally good. Not only does the media (e.g.,^5,6^) support this stereotype, but also the International Federation of Football History & Statistics (IFFHS) and UEFA who give out the most prestigious goalkeeper awards (mainly based on the subjective perception of their judges). German goalkeepers are leading the current list of winners (up until 2020) for all three of these awards (IFFHS World’s Best Goalkeeper: 9, UEFA Best European Goalkeeper: 13, UEFA Best Club Goalkeeper: 6). On the other hand, the English press is particularly critical of their goalkeepers, keeping up the alleged ‘English Goalkeeper Problem’ for years (e.g.,^7–10^). None of the three goalkeeper awards were ever received by an English goalkeeper, a fact that might even negatively influence the public opinion about this stereotype in an additional way. Scientific evidence supporting this public opinion does not exist. If these stereotypes are true, it should be evident in sports performance data. While the performance of a goalkeeper as a whole cannot be easily operationalized, we decided to compare goalkeeping performance as a function of goalkeeper nationality in the standard penalty kick situation. The performance in penalty kicks is an essential aspect for goalkeeping as penalties have always played an important role in major tournaments. They decided the outcome of two FIFA World Cup finals, two UEFA European Championship finals, seven UEFA Champions League finals, and seven UEFA Europa League finals. Penalty kicks will most probably continue to play a crucial role in upcoming events as well, like the 2022 FIFA World Cup.

Scoring a penalty kick seems to be quite easy, especially for a professional football player: The shot is taken from a spot that is only twelve yards away from the goal and no other field player is allowed to interfere in the process. The only obstacle is the opposing goalkeeper for whom it is extremely difficult to successfully guard the net if the penalty is taken with enough force in the right direction. Still, at the highest international level (World Cups and European Championships as well as Champions and Europa League), about 25 percent of all penalty kicks are either saved by the goalkeeper or miss the goal entirely. If there is some truth behind the stereotype that England has relatively bad goalkeepers, it seems reasonable that they would save fewer penalties than goalkeepers from different nations. A brief look at the numbers of the penalty shootouts involving the England national team does in fact show a negative balance. Since the penalty shootout was introduced to major international tournaments in 1976, England has only won three (World Cup in 2018; European Championship in 1996; Nations League in 2019) of the ten shootouts they participated in (losses: World Cups in 1990, 1998 and 2006; European Championships in 1996, 2004, 2012, and 2021). Hence, it might be possible that the players who took the penalty kicks for England were not responsible for this subpar performance^4^, but the English goalkeepers instead. For this reason, this study aims to determine whether the success ratios of goalkeepers in regard to their nationality differ from each other or from the overall average.

Our reasoning for the importance of this research follows that of Brinkschulte, Furley and Memmert^4^, by pointing out that there is evidence showing the impact of stereotypes on performance in sports, even if those stereotypes are not true. A phenomenon that can be considered as being of particular relevance in this regard is the so-called *stereotype threat*^2^. Research on this phenomenon indicates that simply introducing a negative stereotype about a social group can lead to a decrease in the performance of members of that group. In the domain of sports, various studies were able to show that stereotypes can negatively influence athletic performance^11,12^. An alleged stereotype could, for example, not only have an inhibiting effect on learning a new motor skill^13^, but it could also lead to an athlete simply not trying their best^14^. For this reason, we see it as theoretically possible that the continued existence of the stereotype of the ‘English goalkeeper problem’ could potentially have negative consequences as the mere knowledge about this stereotype might contribute to English goalkeepers underperforming (when facing penalties or during matches in general) in the sense of a self-fulfilling prophecy (as indicated in^2,11,12^).

In conclusion, we believe that it is important to examine if English goalkeepers per se perform poorly when facing penalty kicks. If they do indeed perform poorly, a subpar performance of English goalkeepers should be apparent when comparing their success rates with the ones of goalkeepers from other countries. Opposing results would suggest that English goalkeepers are not responsible for England’s poor performance in important penalty shootouts in the past and, in addition, provide a counterargument to the prevalent stereotype of ‘England having a goalkeeper problem’ as stated in the media. To find out, we evaluated the success rates of goalkeepers in penalty kicks they faced in European Championships and World Cups as well as on the highest club level in the UEFA Champions League and the UEFA Europa League.

## Method

Our methodological approach followed that of Brinkschulte et al.^4^. First, we sampled all penalty kicks taken in penalty shootouts and during the matches in European and World Cup competitions since 1976. Second, we sampled all penalty kicks taken in shootouts and during the matches in the UEFA Champions League since the 2000/01 season (there is no reliable data available for penalty kicks taken before the 2000/01 season) and in the UEFA Europa League since the 2004/05 season (the group stage was introduced in the 2004/05 season). These four tournaments were selected because they represent the highest level of professional football in the world, with only the very best players and goalkeepers participating in them. According to several web-based sources (fifa.com, football-coefficient.eu, forbes.com, uefa.com, statista.com), there is not only considerable prize money distributed between the participating teams depending on their performance in these tournaments (total prize money 2021 UEFA European Championship: about €330 million, 2018 FIFA World Cup: about €675 million, 21/22 UEFA Champions League: about €2 billion, 21/22 UEFA Europa League: about €465 million), but these matches also draw the attention of an incredible number of fans who cheer for their nations and favorite clubs. The cumulative viewership of the 2021 UEFA European Championship was 5.2 billion with 328 million people watching the final between England and Italy, and the cumulative viewership of the 2018 FIFA World cup was 3.6 billion with 1.1 billion people watching the final between Croatia and France.

We collected the data from various websites that provide information on the different competitions such as type of tournament and the year it took place, goalkeeper names and their nationalities, successful and unsuccessful penalty kicks taken in shootouts and during the game (e.g., soccerstats.com, wikipedia.org, worldfootball.net, fifa.com, uefa.com, transfermarkt.de). Along with listing the goalkeepers’ names and the dates of the seasons, we coded each penalty kick as either saved or scored. The coded penalties were double-checked using multiple sources. We then used this information to calculate the success rate for each goalkeeper. Additionally, we coded the nationality of all goalkeepers. The independent variable was the nationality of the goalkeeper. The dependent variable was the success rate (percentage of saved penalty kicks plus the additional penalties that hit the post/crossbar or missed the goal) of the respective goalkeeper. As, theoretically, no professional football player would hit the goal post/crossbar or miss the goal entirely when taking an important penalty kick without a goalkeeper guarding the net, these events were considered goalkeeper success as well. In all tournaments, we distinguished between performance in penalty shootouts and performance in in-game penalties. We used the same nations as previous research on cross-national penalty analyses^4^. In the analysis, we directly compared the success rates of goalkeepers from nations that, based on the official FIFA national team ranking (of September 2021, www.fifa.com), are some of the world’s best in football: England (rank 3), Germany (14), Spain (8), Italy (5), Netherlands (11), Brazil (2), Argentina (6), and France (4). These are also the nations with the most appearances in finals of the FIFA World Cup since 1930 and the UEFA European Championship since 1960 (England: 1 World Cup final/1 European Championship final, Germany: 8/6, Spain: 1/4, Italy: 6/4, Netherlands: 3/1, Brazil: 7, Argentina: 5, and France: 3/3). In the 16 UEFA European Championship finals, only two were played without the participation of at least one of these teams (1960 and 2004). None of the 21 FIFA World Cup finals were ever played without the participation of at least one of these teams. As a result, these nations are amongst the countries with the highest number of goalkeepers in the analyzed competitions. The success rates of goalkeepers from all remaining nations were taken together and consolidated into ‘other’. The dataset including all penalty kicks considered for the different analyses of this study is made publicly available as an online supplement.

Since the success rates of the goalkeepers among the individual penalties faced follow a binomial distribution, a generalized linear model (more specifically, a binomial regression) was fit to the data. All analyses were performed using the statistical software R^15^. Five models were fit, (1) for penalty shootouts and (2) in-game penalties in World Cups and European Championships, (3) for penalty shootouts and (4) in-game penalties in the Champions and Europa Leagues, and (5) for all penalty kicks combined across all four tournaments. In each model, the response was modeled as the number of penalties not resulting in a goal (i.e., goalkeeper success) out of the total number of penalties faced by a goalkeeper. The goalkeeper’s nationality was used as the predictor variable. The effect of the goalkeeper’s nationality on the success rate was examined by Wald z-tests of the coefficients as well as by inspecting the odds ratios. The odds ratios indicate how much the success rate of a goalkeeper from a respective nation changes when facing a penalty kick compared to goalkeepers from the other nations. Furthermore, in each model the main effect of nationality on the success rate was examined using Chi-Squared Likelihood Ratio Tests.

## Results

### World Cups and European championships

#### Penalty shootouts

Within penalty shootouts, 71 different goalkeepers faced 473 penalties. On average, the goalkeeper success rate was 26.29 percent (*SD* = 21.51). The Likelihood Ratio Test did not reveal a significant main effect of nationality (χ^2^ [8] = 11.004, *p* = 0.202). Table 1 shows the coefficients for the different nationalities in the binomial regression model including *p*-values of the Wald z-tests and odds ratios.

**Table 1:** Coefficients for the different nationalities in the binomial regression model for all kicks taken in penalty shootouts faced by goalkeepers during World Cup and European Championship matches (123 of 473 penalty kicks were missed against 71 different goalkeepers in the analyzed time periods).

Country	Estimate	SE	z	*p*	Odds Ratio
Other (intercept)	−1.066	0.161	−6.630	< 0.001	0.344
England	−0.202	0.410	−0.494	0.622	0.817
Germany	0.412	0.378	1.091	0.275	1.510
Spain	−0.187	0.432	−0.432	0.666	0.830
Italy	−0.033	0.370	−0.088	0.930	0.968
Netherlands	−0.657	0.511	−1.284	0.199	0.519
Brazil	0.961	0.487	1.973	0.048*	2.613
Argentina	0.624	0.457	1.367	0.172	1.867
France	−0.400	0.481	−0.833	0.405	0.670

The *p* values marked bold represent significant results (*p* < 0.05).

#### In-game penalties

Within in-game penalties, 142 different goalkeepers faced 237 penalties. On average, the goalkeeper success rate was 19.18 percent (*SD* = 33.40). The Likelihood Ratio Test did not reveal a significant main effect of nationality (χ^2^ [8] = 6.029, *p* = 0.644). Table 2 shows the coefficients for the different nationalities in the binomial regression model including *p*-values of the Wald z-tests and odds ratios.

**Table 2:** Coefficients for the different nationalities in the binomial regression model for all in-game penalty kicks faced by goalkeepers during World Cup and European Championship matches (48 of 237 penalty kicks were missed against 142 different goalkeepers in the analyzed time periods). In the analyzed time periods, Brazilian goalkeepers saved none of the in-game penalty kicks they faced.

Country	Estimate	SE	z	*p*	Odds Ratio
Other (intercept)	−1.459	0.203	−7.196	< 0.001	0.233
England	0.206	0.827	0.249	0.803	1.229
Germany	0.360	0.612	0.588	0.556	1.433
Spain	0.948	0.758	1.251	0.211	2.580
Italy	1.053	0.677	1.557	0.120	2.867
Netherlands	0.072	0.816	0.089	0.929	1.075
Brazil	−15.444	1419.487	−0.011	0.991	0.000
Argentina	−0.333	1.099	−0.303	0.762	0.717
France	−0.333	0.790	−0.422	0.673	0.717

**Figure 1:**
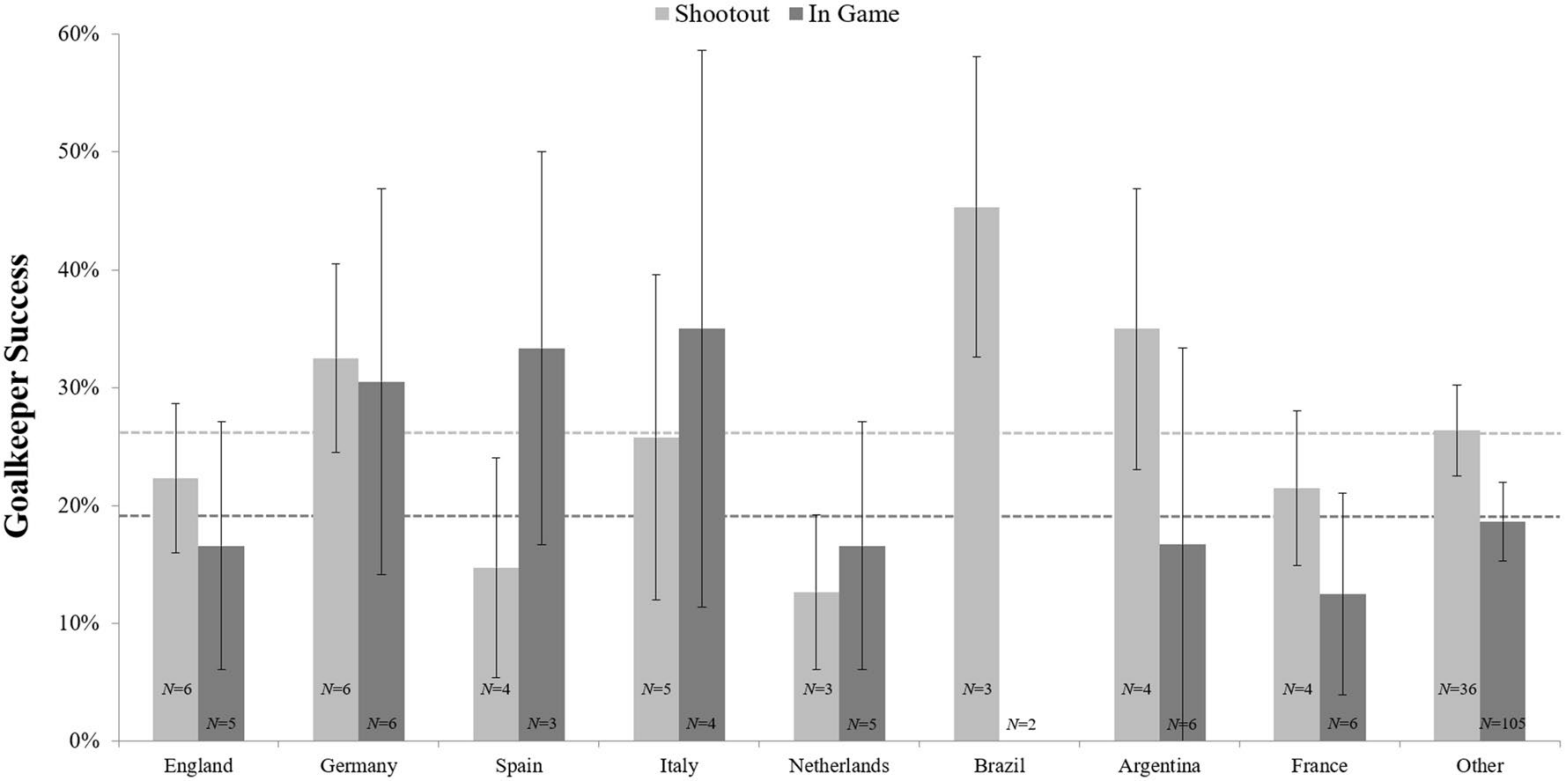
Mean percentages of the goalkeeper success ratios for penalty kicks faced during World Cup and European Championship matches as a function of goalkeeper nationality and type of penalty kick. The *N*’s refer to the number of goalkeepers analyzed for each nation (171 of 710 penalty kicks were missed against 165 different goalkeepers in the analyzed time period). Error bars represent standard errors of the mean.

The goalkeeper performance (success rate) in World Cups and European Championships as a function of nationality and type of penalty kick (shootout vs. in-game) is shown in Fig. 1.

### Champions league and Europa league

#### Penalty shootouts

Within penalty shootouts, 48 different goalkeepers faced 311 penalties. On average, the goalkeeper success rate was 27.95 percent (*SD* = 19.58). The Likelihood Ratio Test did not reveal a significant main effect of nationality (χ^2^ [7] = 8.534, *p* = 0.288). Table 3 shows the coefficients for the different nationalities in the binomial regression model including *p*-values of the Wald z-tests and odds ratios.

**Table 3:** Coefficients for the different nationalities in the binomial regression model for all kicks taken in penalty shootouts faced by goalkeepers during Champions League and Europa League matches (85 of 311 penalty kicks were missed against 48 different goalkeepers in the analyzed time periods). In the analyzed time periods, no penalty kicks were taken against Argentinian goalkeepers in penalty shootouts in the Champions and Europa League.

Country	Estimate	Std. Error	z	*p*	Odds Ratio
Other (intercept)	−1.006	0.213	−4.712	< 0.001	0.366
England	−0.786	1.101	−0.714	0.475	0.456
Germany	0.657	0.433	1.517	0.129	1.929
Spain	0.165	0.333	0.495	0.620	1.179
Italy	0.600	0.937	0.640	0.522	1.822
Netherlands	−1.074	0.648	−1.656	0.098	0.342
Brazil	0.067	0.447	0.150	0.880	1.070
Argentina	–	–	–	–	–
France	−0.198	0.512	−0.388	0.698	0.820

#### In-game penalties

Within in-game penalties, 512 different goalkeepers faced 1,358 penalties. On average, the goalkeeper success rate was 23.47 percent (*SD* = 32.02). The Likelihood Ratio Test did not reveal a significant main effect of nationality (χ^2^ [8] = 7.601, *p* = 0.473). Table 4 shows the coefficients for the different nationalities in the binomial regression model including *p*-values of the Wald z-tests and odds ratios.

**Table 4:** Coefficients for the different nationalities in the binomial regression model for all in-game penalty kicks faced by goalkeepers during Champions League and Europa League matches (341 of 1,358 penalty kicks were missed against 512 different goalkeepers in the analyzed time periods).

Country	Estimate	SE	z	*p*	Odds Ratio
Other (intercept)	−1.083	0.082	−13.221	< 0.001	0.338
England	0.524	0.451	1.162	0.245	1.688
Germany	0.228	0.214	1.342	0.180	1.333
Spain	0.040	0.224	0.179	0.858	1.041
Italy	−0.359	0.319	−1.125	0.261	0.698
Netherlands	−0.039	0.318	−0.122	0.903	0.962
Brazil	−0.359	0.319	−1.125	0.261	0.698
Argentina	0.139	0.453	0.307	0.759	1.149
France	−0.276	0.270	−1.023	0.306	0.759

**Figure 2:**
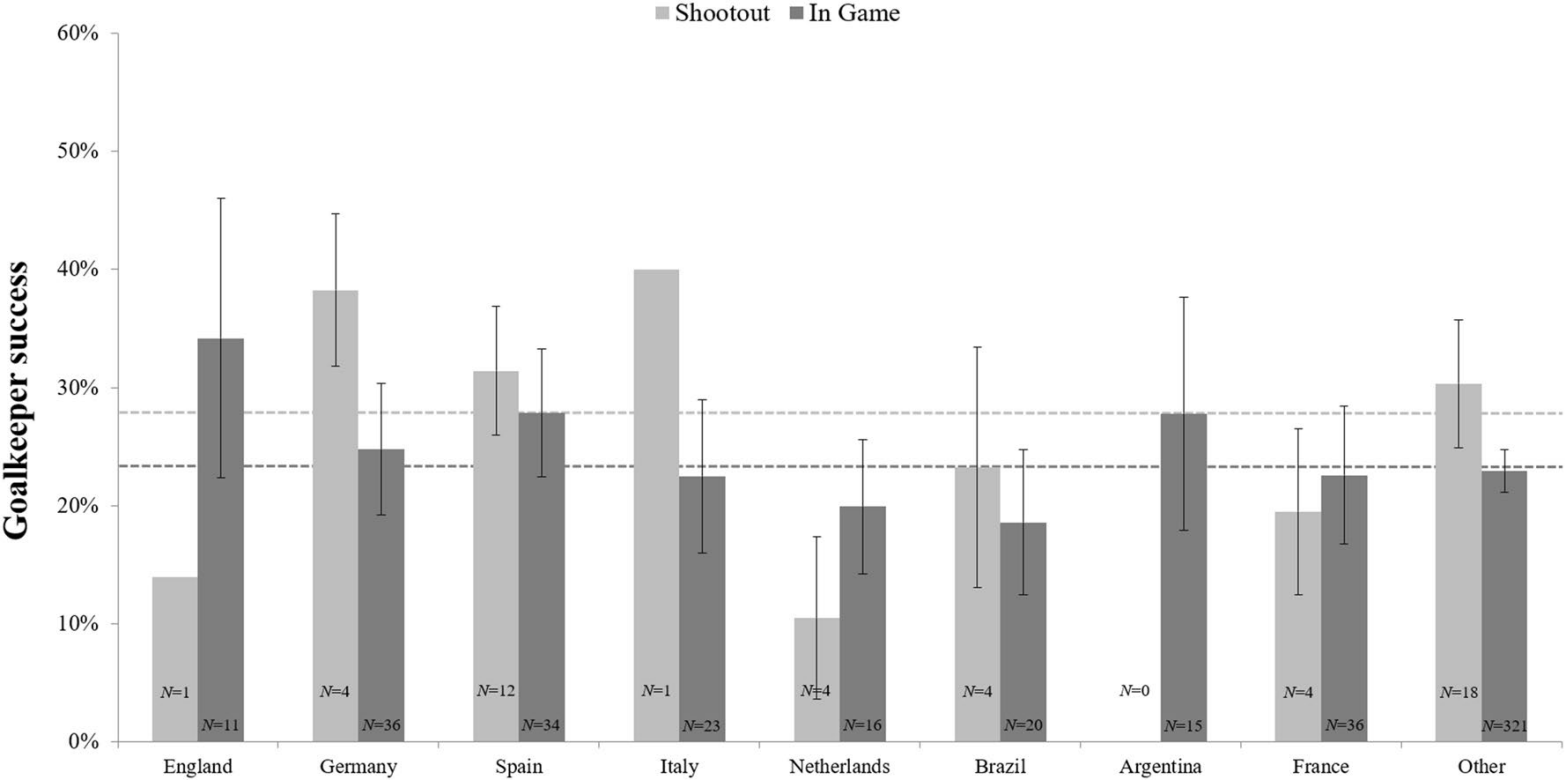
Mean percentages of the goalkeeper success ratios for penalty kicks faced during Champions League and Europa League matches as a function of goalkeeper nationality and type of penalty kick. The *N*’s refer to the number of goalkeepers analyzed for each nation (426 of 1669 penalty kicks were missed against 515 different goalkeepers in the analyzed time periods). Error bars represent standard errors of the mean.

The goalkeeper performance (success rate) in Champions and Europa League matches as a function of nationality and type of penalty kick (shootout vs. in-game) is shown in Fig. 2.

### Combined analysis collapsed over all penalties

Across all four tournaments, 629 different goalkeepers faced a total of 2,379 penalty kicks. On average, the goalkeeper success rate was 22.25 percent (*SD* = 29.89). The Likelihood Ratio Test did not reveal a significant main effect of nationality (χ^2^ [8] = 12.7, *p* = 0.123). Table 5 shows the coefficients for the different nationalities in the binomial regression model including *p*-values of the Wald z-tests and odds ratios.

**Table 5:** Coefficients for the different nationalities in the binomial regression model for all penalty kicks (shootouts and in-game) faced by goalkeepers during World Cup, European Championship, Champions League, and Europa League matches (597 of 2,379 penalty kicks were missed against 629 different goalkeepers in the analyzed time periods).

Country	Estimate	SE	z	*p*	Odds Ratio
Other (intercept)	−1.117	0.065	−17.093	< 0.001	0.327
England	0.035	0.267	0.131	0.896	1.035
Germany	0.394	0.164	2.406	0.016*	1.482
Spain	0.128	0.160	0.799	0.424	1.137
Italy	−0.055	0.216	−0.253	0.800	0.947
Netherlands	−0.340	0.236	−1.441	0.149	0.712
Brazil	0.029	0.218	0.133	0.895	1.029
Argentina	0.312	0.299	1.044	0.296	1.367
France	−0.270	0.205	−1.313	0.189	0.764

The *p* values marked bold represent significant results (*p* < 0.05).﻿

**Figure 3:**
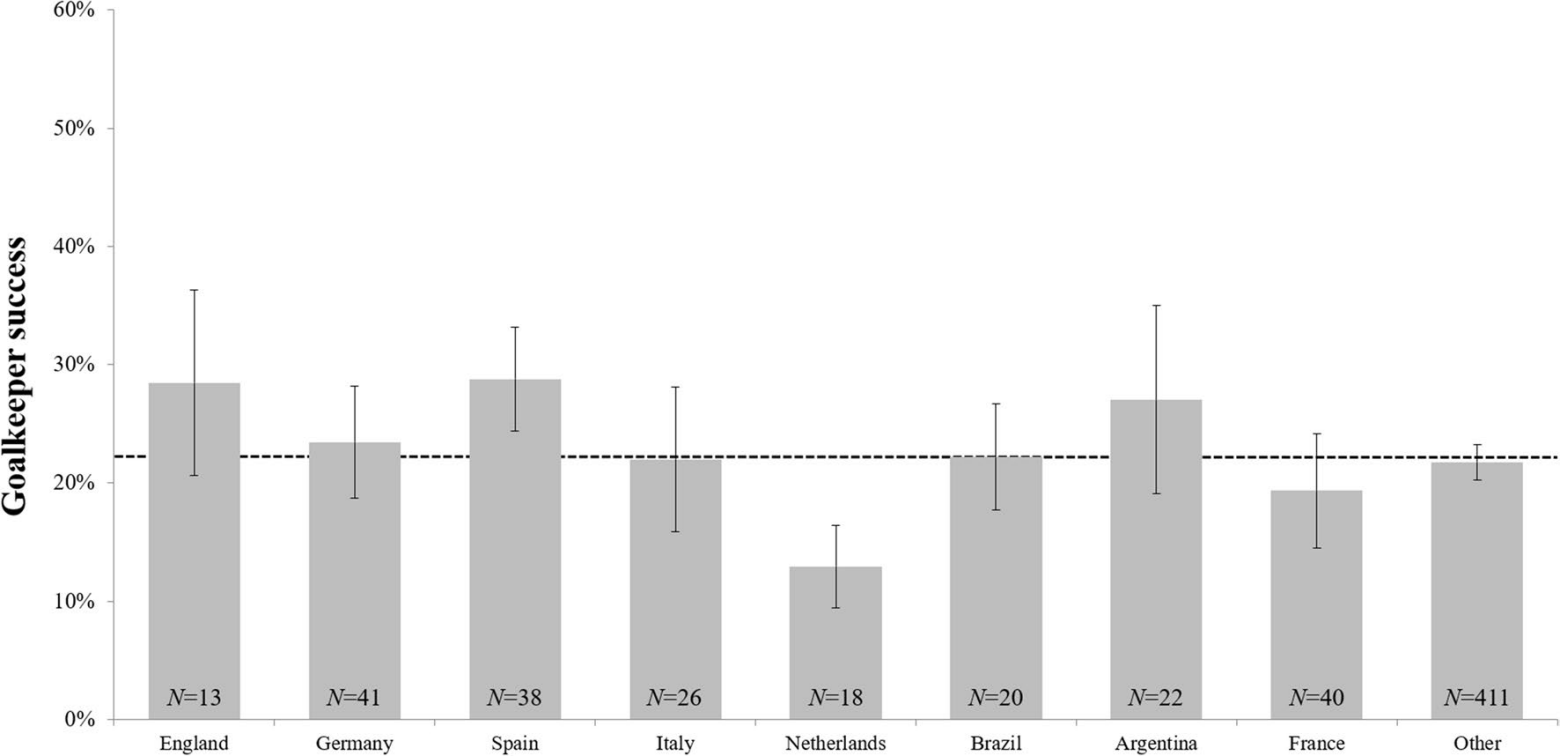
Mean percentages of the goalkeeper success ratios for penalty kicks faced (shootouts and in-game) during World Cup, European Championship, Champions League and Europa League matches as a function of goalkeeper nationality. The *N*’s refer to the number of goalkeepers analyzed for each nation (597 of 2379 penalty kicks were missed against 629 different goalkeepers in the analyzed time periods). Error bars represent standard errors of the mean.

The goalkeeper performance (success rate) in penalty kicks faced during World Cups and European Championships as well as during Champions and Europa League matches as a function of nationality is shown in Fig. 3.

## Discussion

The present research aimed to investigate if the nationality of goalkeepers has an influence on goalkeepers’ success rates of saving penalty kicks. If this were the case and the data showed that English goalkeepers per se underperform in penalty kicks, the results would not only provide a possible explanation for England’s poor performance in important penalty shootouts in the past, but also potential evidence for the alleged ‘goalkeeper problem’. However, the empirical data did not support either of these two stereotypes. Concerning our main research question, the success rate of English goalkeepers was as high as the success rate of goalkeepers from other nations. Linking the prevalence of lost penalty shootouts by the England men’s national team to the nationality of the goalkeepers would therefore be incorrect. Our results indicate that there are no significant differences between the success rates of goalkeepers from different nations (except for German goalkeepers, performing slightly better than the population average), which, in turn, can be considered one counterargument against the stereotype that ‘English goalkeepers are no good’^7–10^.

At a descriptive level of analysis, the average success rate of English goalkeepers in World Cups and European Championships is lower for in-game penalty kicks (16.67%) than for shootouts (22.38%). In both cases, their average success rates are slightly below the respective total sample means for in-game penalties (19.18%) and shootouts (26.29%). In the Champions and Europa League, the average success rate of English goalkeepers in shootouts (14.29%) is second to the lowest among the analyzed nations (total sample mean: 27.95%) but it must be noted that only one English goalkeeper participated in shootouts in the considered time periods (Paul Robinson lost with Tottenham Hotspur against PSV Eindhoven in the round of eight of the 2007/08 Europa League season). For in-game penalty kicks, on the other hand, England’s goalkeepers have the highest average success rate (34.24%) compared to the other nations considered in the analysis and are above the total sample mean of 23.47%. When taking all penalty kicks into account (shootouts and in-game during all four tournaments), the average success rate of English goalkeepers (28.45%) is second to the highest (right behind Spain with an average success rate of 28.75%) and, therefore, above the total sample mean success rate of 22.25%. Either way, the results of the generalized linear models do not indicate a significant main effect of nationality in any of the categories examined. The success rates of goalkeepers from the Netherlands are below average in all categories except for in-game penalties in World cups and European Championships (here, the odds ratio of 1.075 is marginally higher than average) but none of these results reach statistical significance. With an odds ratio of 2.613, the success rate of Brazilian goalkeepers in penalty shootouts in World Cups and European Championships is significantly higher than average. This result is the consequence of a total of nine penalty kicks saved (out of 19, success rate: 47.37%) by Brazilian goalkeepers in shootouts, whereas they did not save any of the four in-game penalty kicks faced in these competitions. Considering in-game penalties and shootouts across all four tournaments, German goalkeepers have a significantly higher success rate compared to goalkeepers from other nations which is reflected by an odds ratio of 1.482. However, when differentiating between the type of penalty kick and the type of competition, this result is not apparent anymore (which might change in the future with a higher number of penalty kicks taken against German goalkeepers). The success rates of England’s goalkeepers in penalty shootouts with odds ratios of 0.817 (World Cups and European Championships) and 0.456 (Champions and Europa League) are below average. In contrast, their success rates for in-game penalties in World Cups and European Championships (odds ratio: 1.229) as well as in the Champions and Europa League (odds ratio: 1.688, category highest) are above average. However, also none of these results reach statistical significance. Considering all these results, we conclude that there are no meaningful differences in the performance of goalkeepers based on their nationality. The reasons for the poor performance in penalty shootouts of the England national team in the past most probably lie with several different factors—including the enormous external pressure when it comes to this crucial moment at the end of an important match as a possible result of English fans expecting their team to finally “bring it home” (win the trophy) as well as the anticipation of the media’s brutal coverage^16^ if success is not achieved. Furthermore, the quite negative public perception of England’s performance in penalty kicks may be the consequence of an unreliable measurement of penalty performance^17^ and the public’s general tendency of stereotyping^18^ in everyday life and in the context of sports^19,20^.

When taking a closer look at the descriptive results of the Champions- and Europa League, however, English goalkeepers stand out in a different aspect: With only twelve goalkeepers, England has the smallest number of goalkeepers who faced penalty kicks among the analyzed nations in the considered time periods. This raises the question of whether teams with English goalkeepers simply commit fewer fouls in the penalty box or if there are fewer English goalkeepers playing for clubs who participate in the Champions and Europa League in general. The latter would mean that the very best clubs in European football prefer playing goalkeepers from other nations over goalkeepers from England. This seems to be tentatively supported by the fact that only 15 different English goalkeepers started for the teams in the Champions League between 2000/01 and 2019/20. From the seven other analyzed nations, only Argentina had fewer (ten goalkeepers) in this time period. In addition, the English goalkeepers only played an average of 8.00 matches (again lowest of the seven analyzed nations), meaning that their clubs either got knocked out of the tournament relatively early or that they switched to goalkeepers from different nations for most of their games. In contrast, Spain (37 starting goalkeepers with an average of 19.35 matches played) and Germany (31 starting goalkeepers with an average of 19.81 matches played) could be considered the nations with the most desirable goalkeepers based on their playing time in the Champions League within the last 20 years. However, when taking the average number of goals scored against goalkeepers from the different nations per match as a proxy for performance, English goalkeepers again did not perform worse (1.43 goals/game) than Spanish (1.20 goals/game), German (1.45 goals/game), Italian (1.49 goals/game), French (1.42 goals/game), Brazilian (1.17 goals/game), Dutch (1.83 goals/game) or Argentinian goalkeepers (1.29 goals/game).

Investigating the reasons behind the lack of English goalkeepers in the Champions League goes beyond the scope of the present study. However, the results of our study indicate that the nationality of a goalkeeper per se does not have an influence on their success rate in penalties and that English goalkeepers are not responsible for England’s poor performance in penalty shootouts in the past. Considering this result, we provide a counterargument against the prevalent stereotype that ‘English goalkeepers are no good’. In order to hold evidence-based results against the negative public opinion regarding English goalkeeping, future research should investigate other possible reasons behind the alleged ‘goalkeeper problem’—the performance of English goalkeepers in penalty kicks is at least not a factor that should contribute to this stereotype.

## Availability of materials and data

The dataset generated and analyzed during the current study as well as an additional statistical approach for data analysis will be made available as online supplements.

## Supplementary Information

Supplementary Information 1.

Supplementary Information 2.

## References

[1] McFadden, B. R. Examining the gap between science and public opinion about genetically modified food and global warming. *PloS One.* **11**(11), e0166140 (2016). 10.1371/journal.pone.0166140 PMC5102371 27829008

[2] Steele CM, Aronson J (1995). Stereotype threat and the intellectual test performance of African Americans. J. Pers. Soc. Psychol..

[3] Furley, P. & Dicks, M. “White men can't jump.” But can they throw? Social perception in European basketball. *Scand. J. Med. Sci Spor*. **24**(5), 857–867 (2014). 10.1111/sms.12086 23714161

[4] Brinkschulte M, Furley P, Memmert D (2020). English football players are not as bad at kicking penalties as commonly assumed. Sci. Rep..

[5] Lyttleton, B. *Twelve yards: The art and psychology of the perfect penalty kick* (Penguin, 2015).

[6] SportSkeeda. Which National Team has the Best Goalkeepers at the Moment? *SportSkeeda* (2020). Available at: https://www.sportskeeda.com/football/which-nation-best-goalkeepers. Accessed 29th March 2021

[7] Eurosport. Are England Actually Bad at Taking Penalties - Or are the Goalkeepers to Blame? Eurosport (2018). Available at: https://www.theguardian.com/football/2007/aug/25/newsstory.sport16. Accessed 29 March 2021

[8] Mirror. England’s No.1 Problem has Happened Again - The Poison Chalice of Being Three Lions’ Goalkeeper. Mirror (2018). Available at: https://www.mirror.co.uk/sport/football/news/englands-no1-problem-happened-again-11770653. Accessed 29 March 2021

[9] The Guardian. What is Wrong with England’s Goalkeepers? The Guardian (2007). Available at: https://www.theguardian.com/football/2007/aug/25/newsstory.sport16. Accessed 29th March 2021

[10] The Guardian. The Secret Footballer: Why is England Lacking in Top Goalkeepers? The Guardian (2011). Available at: https://www.theguardian.com/football/blog/2011/sep/02/secret-footballer-england-goalkeepers. Accessed 29th March 2021

[11] Beilock SL, Jellison WA, Rydell RJ, McConnell AR, Carr TH (2006). On the causal mechanisms of stereotype threat: Can skills that don't rely heavily on working memory still be threatened?. Pers. Soc. Psychol. B..

[12] Stone J, Lynch CI, Sjomeling M, Darley JM (1999). Stereotype threat effects on black and white athletic performance. J. Pers. Soc. Psychol..

[13] Heidrich C, Chiviacowsky S (2015). Stereotype threat affects the learning of sport motor skills. Psychol. Sport Exerc..

[14] Beilock SL, McConnell AR (2004). Stereotype threat and sport: can athletic performance be threatened?. J. Sport Exerc. Psychol..

[15] R Development Core Team. R: A language and environment for statistical computing. *R Foundation for Statistical Computing, Vienna, Austria* https://www.R-project.org/ (2019).

[16] Neville, G. England players are terrified and live in fear of failure. *Daily Mail* (2011). Available at: https://www.dailymail.co.uk/sport/football/article-2028286/Gary-Neville-England-players-terrified-live-fear-failure.html. Accessed 27th Sept 2021

[17] Schweizer G, Furley P, Rost N, Barth K (2020). Reliable measurement in sport psychology: the case of performance outcome measures. Psychol. Sport Exerc..

[18] Fiske ST, Taylor SE (1991). Social cognition.

[19] Buffington D, Fraley T (2011). Racetalk and sport: the color consciousness of contemporary discourse on basketball. Sociol. Inq..

[20] Stone J, Perry ZW, Darley JM (1997). “White men can’t jump”: Evidence for the perceptual confirmation of racial stereotypes following a basketball game. Basic Appl. Soc. Psychol..

