# Supplementary material for: English goalkeepers are not responsible for England’s poor performance in penalty shootouts in the past
Source: Sci Rep. 2021 Dec 28;11:24469. doi: 10.1038/s41598-021-04118-6 (PMC8714815; doi:10.1038/s41598-021-04118-6)
Supplement: Supplementary file 1 — Supplementary Information 1. [file 41598_2021_4118_MOESM1_ESM.docx]

**English Goalkeepers Are Not Responsible for England’s Poor Performance in Penalty Shootouts in the Past**

**– Additional Analysis: Analysis of Variances –**

#### **Method – Analysis of Variances**

In addition to the generalized linear models, in accordance with a previous publication on this topic [1], data were also analyzed with univariate ANOVAs and Bonferroni corrected pairwise comparisons. The level of significance was set at 0.05. Partial eta-squared effect sizes were calculated and interpreted in line with statistical convention as 0.01 being a small effect, 0.06 a medium effect, and 0.14 a large effect. In addition, we calculated one-sample t-tests comparing the performance of every nation to the mean of the entire sample. If assumptions of the parametric tests were violated, we conducted non-parametric tests (i.e., Mann-Whitney-U-Tests). Inferential statistics are reported for both success rates (i.e., percentages of saved plus missed penalties) and percentages of saved penalties only (i.e., excluding penalties that hit the post/crossbar or missed the goal). In all tournaments, we distinguished between performance in penalty shootouts and performance in in-game penalties.

#### **Results**

**World Cups and European Championships.**

***Penalty shootouts.*** Within penalty shootouts, 71 different goalkeepers faced 473 penalties. On average, the goalkeeper success rate was 26.29 percent (*SD* = 21.51; 13.55 for percent saved; *SD* = 15.90). A univariate ANOVA revealed neither a main effect for the factor nationality on the goalkeeper success rates (*F*[8, 62] = 0.752, *p* = .646, *η*² = 0.088) nor on the percentages saved (*F*[8, 62] = 1.369, *p* = .228, *η*² = 0.150). Although the effect was non-significant, it can be considered a medium effect for the total success rate and a large effect for the percentage of saved penalties by statistical convention. None of the Bonferroni corrected pairwise comparisons revealed significant differences between any of the analyzed nations (all *p* > .99). Hence, there were no significant differences between the success rates or percentages saved of goalkeepers from different nations. A Kruskal-Wallis test for the nine different nation categories confirmed this non-significant effect on goalkeeper success rates (χ2 = 7.764, df = 8, *p* = .457) and on percentages saved (χ2 = 10.476, df = 8, *p* = .233). None of the nations significantly (all *p* > .179, two-tailed) differed from the overall sample mean success rate of 26.29 percent nor from the overall sample mean of 13.55 percent saved penalties (all *p* > .166; except for the Netherlands who saved significantly less penalties: 1.67 %, *SD* = 2.88, *p* = .019).

***In-game penalties.*** Within in-game penalties, 142 different goalkeepers faced 237 penalties. On average, the goalkeeper success rate was 19.18 percent (*SD* = 33.40; 8.41 for percent saved; *SD* = 23.16). A univariate ANOVA revealed neither a main effect for the factor nationality on the goalkeeper success rates (*F*[8, 133] = 0.376, *p* = .932, *η*² = 0.022) nor on the percentages saved (*F*[8, 133] = 0.222, *p* = .987, *η*² = 0.013). Although the effect was non-significant, it can be considered a small effect for both the total success rate and the percentage of saved penalties by statistical convention. None of the Bonferroni corrected pairwise comparisons revealed significant differences between any of the analyzed nations (all *p* > .99). Hence, there were no significant differences between the success rates of goalkeepers from different nations. A Kruskal-Wallis test for the nine different nation categories confirmed this non-significant effect on goalkeeper success rates (χ2 = 4.668, df = 8, *p* = .792) and on percentages saved (χ2 = 4.166, df = 8, *p* = .842). None of the nations significantly (all *p* > .469, two-tailed) differed from the overall sample mean success rate of 19.18 percent nor from the overall sample mean of 8.41 percent saved penalties (all *p* > .544). The goalkeeper performance (success rate) in World Cups and European Championships as a function of nationality and type of penalty kick (shootout vs. in-game) is shown in Figure 1 of the main manuscript.

**Champions League and Europa League.**

***Penalty shootouts.*** Within penalty shootouts, 48 different goalkeepers faced 311 penalties. On average, the goalkeeper success rate was 27.95 percent (*SD* = 19.58; 20.35 for percent saved; *SD* = 18.23). A univariate ANOVA revealed neither a main effect for the factor nationality on the goalkeeper success rates (*F*[7, 40] = 0.944, *p* = .484, *η*² = 0.142) nor on the percentages saved (*F*[7, 40] = 0.734, *p* = .644, *η*² = 0.114). Although the effect was non-significant, it can be considered a large effect for the total success rate and a medium effect for the percentage of saved penalties by statistical convention. None of the Bonferroni corrected pairwise comparisons revealed significant differences between any of the analyzed nations (all *p* > .99). Hence, there were no significant differences between the success rates of goalkeepers from different nations. A Kruskal-Wallis test for the nine different nation categories confirmed this non-significant effect on goalkeeper success rates (χ2 = 7.124, df = 7, *p* = .416) and on percentages saved (χ2 = 6.079, df = 7, *p* = .531). None of the nations significantly (all *p* > .085, two-tailed) differed from the overall sample mean success rate of 27.95 percent nor from the overall sample mean of 20.35 percent saved penalties (all *p* > .192; except for the Netherlands who saved significantly less penalties: 6.75 %, *SD* = 7.81, *p* = .040).

***In-game penalties.*** Within in-game penalties, 512 different goalkeepers faced 1,358 penalties. On average, the goalkeeper success rate was 23.47 percent (*SD* = 32.02; 18.76 for percent saved penalties; *SD* = 29.89). A univariate ANOVA revealed neither a main effect for the factor nationality on the goalkeeper success rates (*F*[8, 503] = 0.372, *p* = .935, *η*² = 0.006) nor on the percentages saved (*F*[8, 503] = 0.945, *p* = .479, *η*² = 0.015). Although the effect was non-significant, it can be considered a small effect for the percentage of saved penalties by statistical convention. None of the Bonferroni corrected pairwise comparisons revealed significant differences between any of the analyzed nations (all *p* > .99). Hence, there were no significant differences between the success rates of goalkeepers from different nations. A Kruskal-Wallis test for the nine different nation categories confirmed this non-significant effect on goalkeeper success rates (χ2 = 3.459, df = 8, *p* = .902) and on percentages saved (χ2 = 7.847, df = 8, *p* = .449). None of the nations significantly (all *p* > .223, two-tailed) differed from the overall sample mean success rate of 23.47 percent nor from the overall sample mean of 18.76 percent saved penalties (all *p* > .222; except for the Netherlands who saved significantly less penalties: 7.44 %, *SD* = 13.61, *p* = .005). The goalkeeper performance (success rate) in Champions and Europa League matches as a function of nationality and type of penalty kick (shootout vs. in-game) is shown in Figure 2 of the main manuscript.

**Combined analysis collapsed over all penalties**

Across all four tournaments, 629 different goalkeepers faced a total of 2,379 penalty kicks. On average, the goalkeeper success rate was 22.25 percent (*SD* = 29.89; 16.83 percent for saved penalties; *SD* = 27.46). A univariate ANOVA revealed neither a main effect for the factor nationality on the goalkeeper success rates (*F*[8, 620] = 0.651, *p* = .734, *η*² = 0.008) nor on the percentages saved (*F*[8, 620] = 0.878, *p* = .535, *η*² = 0.011). Although the effect was non-significant, it can be considered a small effect for the percentage of saved penalties by statistical convention. None of the Bonferroni corrected pairwise comparisons revealed significant differences between any of the analyzed nations (all *p* > .99). Hence, there were no significant differences between the success rates of goalkeepers from different nations. A Kruskal-Wallis test for the nine different nation categories confirmed this non-significant effect on goalkeeper success rates (χ2 = 9.380, df = 8, *p* = .311) and on percentages saved (χ2 = 13.240, df = 8, *p* = .104). None of the nations significantly (all *p* > .143, two-tailed) differed from the overall sample mean success rate of 22.25 percent (except for the Netherlands: 12.98 %, *SD* = 14.91, *p* = .017) nor from the 16.83 percent saved penalties (all *p* > .261; except for the Netherlands who saved significantly less penalties: 6.39 %, *SD* = 13.61, *p* = .001). The goalkeeper performance (success rate) in penalty kicks faced during World Cups and European Championships as well as during Champions and Europa League matches as a function of nationality is shown in Figure 3 of the main manuscript.

The results of the analysis of variance do not noticeably differ from the results of the generalized linear models fit to the data presented in the manuscript. None of the comparisons between the average success rates of goalkeepers from the different analyzed nations reached statistical significance, except that Dutch goalkeepers performed worse than goalkeepers from other nations. However, when taking a look at the results of the generalized linear models in the manuscript, the results for Dutch goalkeepers do not reach significance. Considering these findings, we suggest that the factor nationality hardly explains meaningful variance in goalkeeper success rates in penalty kicks.

#### **References**

1. Brinkschulte, M., Furley, P. & Memmert, D. English Football Players are not as Bad at Kicking Penalties as Commonly Assumed. *Sci. Rep*. **10**(1), 1-5 (2020).
